# Relationship between the Brazilian version of the Montreal-Toulouse language assessment battery and education, age and reading and writing characteristics. A cross-sectional study: Relação entre a bateria Montreal-Toulouse de avaliação da linguagem, versão brasileira, e escolaridade, idade e hábitos de leitura e escrita. Um estudo transversal

**DOI:** 10.1590/1516-3180.2014.8461610

**Published:** 2015-03-17

**Authors:** Karina Carlesso Pagliarin, Gigiane Gindri, Karin Zazo Ortiz, Maria Alice Mattos Pimenta Parente, Yves Joanette, Jean-Luc Nespoulous, Rochele Paz Fonseca

**Affiliations:** I PhD. Postdoctoral student, Speech Pathology and Audiology Department, Universidade Federal de Santa Maria (UFSM), Santa Maria, Rio Grande do Sul, Brazil.; II PhD. Speech Therapist at Hospital Nossa Senhora da Conceição, Porto Alegre, Rio Grande do Sul, Brazil.; III PhD. Speech Therapist and Associate Professor, Speech Pathology and Audiology Department, Universidade Federal de São Paulo (Unifesp), São Paulo, Brazil.; IV PhD. Senior Professor, Psychology Department, Universidade Federal do Rio Grande do Sul (UFRGS), Porto Alegre, Rio Grande do Sul, Brazil.; V PhD. Speech Therapist and Professor, Centre de Recherche de l’Institut Universitaire de Gériatrie de Montréal (CRIUGM), Faculté de Médecine, Université de Montréal, Montreal, Canada.; VI PhD. Emeritus Professor in Language Sciences, Laboratoire Jacques-Lordat, Université de Toulouse-Le Mirail, Toulouse, France.; VII PhD. Speech Therapist, Psychologist and Associate Professor, Psychology Department, Pontifícia Universidade Católica do Rio Grande do Sul (PUCRS), Porto Alegre, Rio Grande do Sul, Brazil.

**Keywords:** Language tests, Educational status, Age factors, Aphasia, Adult, Testes de linguagem, Escolaridade, Fatores etários, Afasia, Adulto

## Abstract

**CONTEXT AND OBJECTIVE:**

There is growing concern about understanding how sociodemographic variables may interfere with cognitive functioning, especially with regard to language. This study aimed to investigate the relationship between performance in the Brazilian version of the Montreal-Toulouse language assessment battery (MTL-BR) and education, age and frequency of reading and writing habits (FRWH).

**DESIGN AND SETTING:**

Cross-sectional study conducted in university and work environments in Rio Grande do Sul, Brazil.

**METHOD:**

The MTL-BR was administered to a group of 233 healthy adults, aged 19 to 75 years (mean = 45.04, standard deviation, SD = 15.47), with at least five years of formal education (mean = 11.47, SD = 4.77).

**RESULTS:**

A stepwise multiple linear regression model showed that, for most tasks, the number of years of education, age and FRWH were better predictors of performance when analyzed together rather than separately. In separate analysis, education was the best predictor of performance in language tasks, especially those involving reading and writing abilities.

**CONCLUSION:**

The results suggested that the number of years of education, age and FRWH seem to influence performance in the MTL-BR, especially education. These data are important for making diagnoses of greater precision among patients suffering from brain injuries, with the aim of avoiding false positives.

## RESUMO

**CONTEXTO E OBJETIVO:**

Há uma preocupação crescente com o entendimento de como as variáveis sociodemográficas podem interferir no funcionamento cognitivo, especialmente na linguagem. Este estudo teve como objetivo investigar a relação entre o desempenho na Bateria Montreal-Toulouse de Avaliação da Linguagem, em sua versão brasileira (MTL-BR), e a escolaridade, a idade e a frequência de hábitos de leitura e escrita (FHLE).

**TIPO DE ESTUDO E LOCAL:**

Estudo transversal conduzido em ambientes universitários e de trabalho do Rio Grande do Sul, Brasil.

**MÉTODOS:**

A Bateria MTL-BR foi administrada em um grupo de 233 adultos saudáveis, de 19 a 75 anos de idade (M = 45,04, desvio padrão, DP = 15,47), com no mínimo cinco anos de estudo formal (M = 11,47, DP = 4,77).

**RESULTADOS:**

O modelo de regressão linear múltipla (*stepwise*) mostrou que, para a maioria das tarefas, os anos de escolaridade, de idade e FHLE são melhores preditores de desempenho quando analisados em conjunto do que separadamente. Quando analisada isoladamente, a escolaridade foi o melhor preditor para o desempenho nas tarefas linguísticas, principalmente nas tarefas que envolvem habilidades de leitura e escrita.

**CONCLUSÃO:**

Os resultados mostraram que os anos de escolaridade, idade e FHLE parecem influenciar o desempenho na MTL-BR, principalmente a escolaridade. Esses dados são relevantes para a realização de diagnóstico mais preciso de pacientes que sofreram lesão cerebral a fim de evitar falsos positivos.

## INTRODUCTION

Interpretations on the findings from neuropsychological assessments of language tend to have significant bias because of difficulty in distinguishing between cognitive effects due to brain damage and biological and sociocultural traits, in patients examined.^1^^,^^2^ Thus, there is growing concern about understanding how age, gender, race, education and socioeconomic status, among other factors, may interfere with cognitive functioning.^3^^,^^4^^,^^5^^,^^6^

Among the abovementioned factors, education level and age are the ones highlighted in the literature as the core influences on cognition. Overall, old age and low education level have been correlated with decreased performance.^7^^,^^8^^,^^9^^,^^10^^,^^11^ However, this relationship is not always linear, given that there may be interactions between these factors, such as in naming and verbal fluency tasks, in which the effect of age is canceled when individuals are highly educated.^12^^,^^13^^,^^14^ In addition, although education is considered to be the cultural factor that has the greatest influence on cognition, there are limitations in analyses on this variable. Education level is generally based on the number of years of study, which addresses differences in the duration and not the quality of education.

One alternative that has been suggested is to use reading level^15^ as a predictor of cognitive function. Since participants who have higher frequency of reading and writing habits (FRWH) are more proficient in these skills and therefore score better in cognitive and language tests,^6^^,^^10^^,^^16^^,^^17^^,^^18^ reading performance has been regarded as equally or more important than education level. It is also worth noting that the practice of writing can influence reading comprehension: people who read more write better and vice versa.^19^^,^^20^

The effects of age and education level on healthy samples have been investigated in Brazilian research studies using the two main language assessment batteries: Boston Diagnostic Aphasia Examination (BDAE)^21^ and Montreal-Toulouse Language Assessment Battery.^22^ These studies have aimed to demonstrate the strong impact of these two factors on tasks involving oral and written comprehension, reading aloud, spelling, naming, written naming, reading numbers and copying. The results have revealed that older individuals with lower education levels are the ones who underperform. However, no studies investigating the independent effects of age, education and FRWH and their interactions in aphasia batteries have been conducted in Brazilian populations.^8^^,^^9^^,^^23^

Several tools designed to assess language among patients with aphasia have been described in the international literature. The BDAE,^21^ Aachen Aphasia Test (AAT)^24^ and Western Aphasia Battery (WAB)^25^ are the ones that stand out. Although not as widely used, another battery of Francophone origin that is used for language assessment is the Montreal-Toulouse Language (MTL) Assessment Battery.^22^ The Brazilian Portuguese adaptation of this battery (MTL-BR)^26^ has been seen to present interesting advantages with regard to evaluation and clinical interpretation of the different components of oral and written language. In particular, this adapted version has made it possible to analyze dissociations between different inputs and outputs, and different levels of complexity (word, sentence and discourse).^26^ The psychometric measurements were verified in a previous study^27^ that found evidence of validity (Cronbach’s alpha 0.79-0.90) and reliability (mean test-retest score of 0.52) for the battery. Nevertheless, there is still a lack of research on healthy participants, with the aim of comprehending the effect of each sociocultural and/or biological factor on linguistic and language-related abilities.

## OBJECTIVE

In this context, this study aimed to investigate the relationship between the Montreal-Toulouse language assessment battery and education, age and FRWH, using a sample of neurologically healthy adults.

## METHODS

### Participants

The study included 233 neurologically healthy adults from southern Brazil: 151 females and 82 males. Their ages ranged from 19 to 75 (mean = 45.04; standard deviation, SD = 15.47) and education level ranged from 5 to 23 years of formal schooling (mean = 11.47; SD = 4.77).

The exclusion criteria included: a) impaired vision and/or hearing that was not corrected by means of visual and/or hearing aids; b) signs suggestive of neurological/psychiatric conditions; c) signs of moderate to severe depression (scores above 19 points), as measured using the Beck Depression Inventory (BDI-II);^28^ and d) signs of cognitive decline, as measured using the clock-drawing test^29^ in association with the Mini-Mental State Examination (MMSE),^30^ with scores below 22 points for individuals with 5 to 8 years of schooling and below 24 points for more than 8 years.^31^ In addition, participants who had a history of alcoholism and/or current or previous abuse of illicit drugs or benzodiazepines and antipsychotics, except for atypical neuroleptics (data collected through a questionnaire on the sociocultural aspects of health),^32^ were not included in the study.

### Materials and procedure

The participants were recruited from university environments and work centers (convenience sample). After receiving clarifications about the study, all participants signed a consent form and participated as unpaid volunteers. It is important to state that all ethical procedures were respected, with a guarantee that participation in the study would be voluntary. The study was conducted under approval by the research ethics committee of a higher-education institution (Pontifical Catholic University of Rio Grande do Sul; under approval no. 04908/09).

The FRWH was evaluated using an inventory that included questions about reading habits (magazines, newspapers, books and other materials) and writing habits (text messages, letters and other materials), and the frequency of each activity was scored as follows: 4 points for every day; 3 for several days a week; 2 for once a week; 1 for rarely; and 0 for never, with a maximum frequency score of 28 points.^33^ In this sample, the 14-point band was regarded as the median. Scores higher and lower than 14 were thus denominated high FRWH or low FRWH, respectively.

Finally, the participants were evaluated using the MTL-BR battery.^26^ This tool enables characterization of the subjects’ oral and written language expression and comprehension behavior. The tasks used for this study were:

1. Directed interview: Includes 13 open-ended questions to analyze speech and auditory comprehension. However, only comprehension is scored, with a maximum score of 26 points: 13 items with maximum score of two points each.
2. Automatic speech: Assesses the ability to evoke automatisms such as numbers, days of the week and the birthday song. This task involves formal factors (occurrences of phonemic errors), which are scored with a maximum of six points, and content factors (occurrences of omissions), also with a maximum score of six points.
3. Auditory comprehension: Measures the ability to identify images that represent words and phrases from auditory input. The task consists of a total of 19 items, five words (plates with six stimuli comprising one target and five distracters: one phonological, one semantic, one visual and two neutral) and 14 sentences (both simple and complex). The maximum score is five points for words and 14 points for phrases, with one point for each correct answer.
4. Oral narrative task: Evaluates the ability to tell a story from visual inputs. The task consists of describing a picture depicting a bank robbery. The narrative is analyzed for the number of words produced and the information units (IU) produced (microstructure): bank, robbery, thieves, guard, car, running, waiting, calling, people and money. Each word gets one IU point. Furthermore, three major elements of the scene (macrostructure) are scored: the robbery itself (main scene), someone waiting for the thieves and someone telling the police. The maximum score for the components of the microstructure (IU) is 10 points and for the macrostructure (main elements of the scene), three points.
5. Written comprehension: Assesses the ability to identify the input from visual images corresponding to words and written sentences. The task consists of a total of 13 items, five words (plates with six stimuli comprising one target and five distracters: one orthographic, one semantic, one visual and two neutral) and eight phrases (both simple and complex). The maximum score is five points for words and eight points for phrases, with one point for each correct answer.
6. Sentence copying: Assesses the ability to recognize and reproduce letters. The task consists of a sentence made of eight words. The maximum score is eight points, with one point for each word spelled correctly. Verbatim or servile copying is not considered to be correct.
7. Dictation: Assesses the individual’s ability to understand the auditory stimulus and search the corresponding written representation. The task consists of nine words (regular, irregular, foreign words and non-words) and two sentences. The maximum score is 22 points, with one point for each word written correctly, including phrases.
8. Repetition: Measures the individual’s ability to reproduce auditory stimulus orally, following verbal models. The task consists of 11 words (regular, irregular and non-words) and three sentences. The maximum scores are 11 and 22 points for words and phrases respectively, with one point for each word produced correctly.
9. Reading: Assesses the ability to recognize letters and produce the appropriate phonological sounds corresponding to 12 words (regular, irregular, foreign words and non-words) and three target sentences. The maximum scores are 12 and 21 points for words and phrases respectively, with one point for each correct answer.
10. Semantic verbal fluency: Evaluates spontaneous production of words in the category “animals” within a time period of 90 seconds. Each word correctly selected from this class is equivalent to one point, ignoring repetitions, morphological derivatives of the same word and other words that do not match the requested category.
11. Naming: Measures the ability to identify and name pictures that refer to nouns and verbs, from a visual input. Fifteen pictures are presented (12 nouns and three actions), placed on individual boards. The maximum score is 30 points, comprising 15 items with a maximum score of two points each.
12. Nonverbal praxis: Assesses the ability to produce isolated gestures and movement sequences involving the face and tongue, requested by the evaluator through verbal instructions. The task consists of a total of six items with maximum scores of four points each, giving a maximum total of 24 points.
13. Object manipulation: Assesses the ability to understand simple and complex commands. The individual is instructed to perform six commands given by the clinician, using physical objects (key, comb, cup, pen and paper). The complexity of orders increases gradually. The maximum score is 16 points.
14. Phonological verbal fluency: Evaluates spontaneous production of words that start with the letter M within a time period of 90 seconds. Each correct word equals one point, ignoring repetitions, morphological derivatives of the same word and proper names.
15. Body part recognition and left-right orientation: Assesses the individual’s ability to identify parts of the body and their laterality. The maximum score is eight points, of which four points are given for each body part (limbs) and the other four are given for the right-left orientation.
16. Written naming: Assesses the ability to identify and indicate 15 figures referring to 12 nouns and three actions (verbs), from visual input. The examiner presents each of the figures on different boards. The maximum score is 30 points, comprising 15 items with a maximum score of two points each.
17. Oral text comprehension: Assesses the ability to understand auditory input from a text read by the clinician. The individual must answer six questions orally or in writing after listening to the text (three open-ended and three closed-ended questions). The maximum score is nine points: a maximum of 2 points for each of the three open-ended questions and one point for each of the closed-ended questions.
18. Number dictation: Assesses the ability to understand the auditory stimulus and write down the corresponding number. The task consists of six numbers. Each number written correctly gets one point, with a maximum score of six points.
19. Reading of numbers: Assesses the ability to recognize numerical and visual stimuli and reproduce them orally. The maximum score is six points: one point for each number read correctly.
20. Written narrative: Involves an individual’s ability to write a story from visual input. The task consists of a picture depicting a robbery at a bakery. We analyzed the number of words produced and information units (IU) produced (microstructure): bakery, robbery, robbers, guard, car, running, waiting, calling and gun (one point for each word). In addition, three major elements of the scene (macrostructure) are scored: the robbery itself (main scene), someone waiting for the bandits and someone telling the police. The maximum score is 10 points for IU and three points for elements of the scene.
21. Written text comprehension: Evaluates the ability to decode and interpret linguistic signs in a written text. The task consists of six questions (three open-ended and three closed-ended) that can be answered orally or in writing after reading. The maximum score is nine points: a maximum score of two points each for the three open-ended questions and one point each for the closed-ended questions.
22. Calculation: Evaluates the ability to perform the numerical operations of addition, subtraction, multiplication and division, as well as mental and written mathematical problems. The maximum score is 12 points: one point for each correctly solved calculation (four mental and four written) and two math problems.

The participants were evaluated in a single 90-minute session, by properly trained and qualified healthcare professionals who had completed or were in the process of completing additional training in language neuropsychology. The MTL-BR score was determined by two reviewers, and the final score was established by consensus.

### Statistical analysis

The data were analyzed using SPSS 17.0. Initially, Pearson correlation analysis was used to investigate the relationship between age, education and FRWH and the scores from the MTL-BR tasks. Next, multiple linear regression analysis using the stepwise method was performed in order to identify which variables provided the best explanatory models. A significance level of P ≤ 0.05 was applied.

## RESULTS

The scores from 17 out of the 22 tasks were significantly correlated with education, while the scores from nine subtests (oral narrative task, written comprehension, dictation, reading, semantic and phonological verbal fluency, written naming, written narrative and calculation) achieved moderate positive correlations. Ten tasks were negatively correlated with age (directed interview, auditory comprehension, oral narrative task total number of words, written comprehension, repetition, semantic verbal fluency, naming, written naming, written narrative total number of words and oral text comprehension). Performance in the oral narrative task, dictation, reading, semantic and phonological verbal fluency, written naming, written narrative and calculation presented significant moderate positive correlation with FRWH. The highest correlation was found between the written narrative task (total number of written words) and education variables, followed by the association between semantic verbal fluency and education (Table 1).

**Table 1.**
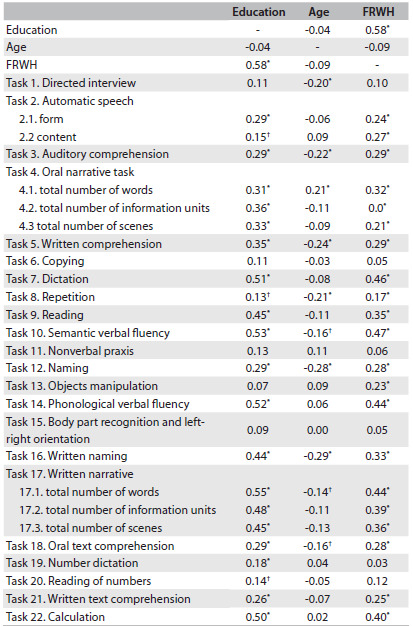
Correlations between education level, age and frequency of reading and writing habits (FRWH) and the tasks of the Montreal-Toulouse language assessment battery, Brazilian version (MTL-BR)

^*^P ≤ 0.001; ^†^P ≤ 0.05.

Table 2 displays the results from the regression analysis on the predictive model for performance in language tasks, considering education, age and FRWH as predictors. As the results show, education was a significant predictor (P < 0.01) for most tasks, with explanatory power ranging from 6% to 28%. Interestingly, it was also the only significant predictive factor in seven subtests: automatisms (form), oral narrative task (total number of IU), reading, number dictation, reading of numbers, written narrative (total number of scenes) and written text comprehension. The FRWH was a significant predictor only for automatisms (content), while age was the only predictor for the guided interview.

**Table 2.**
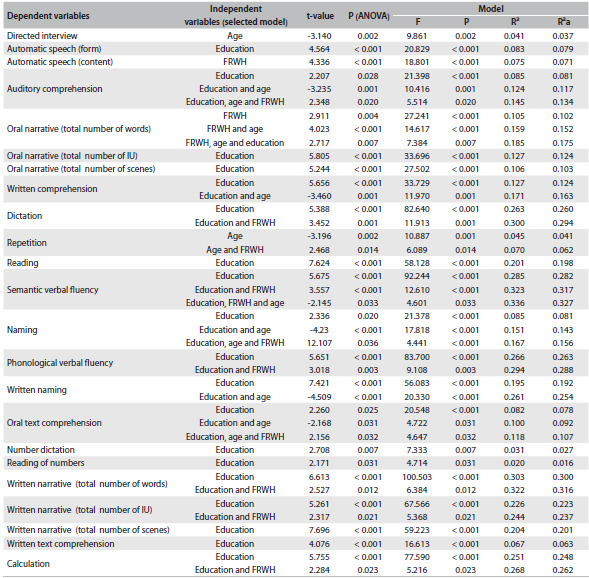
Results from multiple regression analysis on the tasks of the Montreal-Toulouse language assessment battery, Brazilian version (MTL-BR), considering the study variables and the total number of participants

IU = information units; FRWH = frequency of reading and writing habits; ANOVA = analysis of variance; F = F-statistics; P = P-value; R² = coefficient of determination (R squared); R²a = adjusted R squared.

In general, among the tests in which education was combined with other variables, the variable FRWH contributed 2-3% to the explanatory model. In contrast, age contributed 3-6% to the explanatory power when combined with education in some tasks.

With regard to the explanatory models for each dependent variable, education level and FRWH jointly explained 32% of the variance of performance in the semantic verbal fluency and written narrative task (total number of words) and 30% of the variance relating to dictation. Education and age explained 25% of the scores for written naming. These three variables combined showed the greatest explanatory power (33%) for the semantic verbal fluency task. These findings corroborate the results presented in Table 1 (significant correlations).

## DISCUSSION

The results from this study revealed that the MTL-BR is mainly influenced by education, such that higher education level was correlated with better performance. Likewise, other studies have shown the importance of the number of years of formal education in language tasks, with findings similar to those observed in the present study.^1^^,^^6^^,^^8^^,^^9^^,^^10^^,^^33^

As seen in the results, in this study there was a moderate positive correlation with education in tasks that involve dictation, written naming, written comprehension, auditory comprehension and written narrative. These tasks require reading-writing abilities that are developed over the course of education. However, education was not a predictor of the tasks relating to the guided interview, repetition, automatic speech (content) and oral narrative task (total number of words) (Table 2). Previous research has shown that tasks involving graphic stimuli (written comprehension, dictation, reading, written naming, number dictation, reading of numbers, written narrative and written text comprehension) tend to be more sensitive to the influence of education.^6^^,^^8^^,^^11^

The importance, albeit weaker, of FRWH in linguistic performance deserves to be highlighted, given that it increases the predictive power of the education variable. Regularly practicing reading and writing can compensate for low education levels in linguistic performance,^17^^,^^33^ and the quality of what is read is one of the biggest predictors of cognitive performance.^16^^,^^34^ Despite the relevance of FRWH in this study, although this variable had significant positive correlations with various tasks of the MTL-BR (Table 1), it did not show strong explanatory power in comparison with a model composed only by education (Table 2). The main hypothesis for this finding relates to the information obtained from FRWH, since this was restricted to measurement of the frequency of the material that was read and produced, but not the quality, level of complexity or resulting potential for neurocognitive stimulation of this material. However, Pawlowski et al.^33^ used this same questionnaire to verify the influence of FRWH combined with education level on performance in neuropsychological tasks among adults and noted that frequent practice of reading and writing among individuals with low education promoted an improvement in cognitive performance, as well as in language tasks.

The greater explanatory power found in joint education-FRWH models is based on the combination of these two sociocultural variables: the higher the education level and the frequency of reading and writing habits are, the better the scores in language tasks will be. On the other hand, people with low education levels are less motivated to perform tasks that require reading and writing in their day-to-day lives,^17^^,^^35^ which can hamper understanding in some subtests involving increasing complexity and may cause lower performance.

It has been shown that not only education level, but also reading scores are predictors in the verbal fluency test, naming task and comprehension.^10^^,^^17^^,^^18^ This is mainly because anyone who reads and writes more often possesses a richer vocabulary, and makes use of attention, mnemonic and executive skills. People who tend to read less may present cognitive decline and worse outcomes in reading tasks, especially the elderly.

The habits of reading and writing are also considered to be one of the factors that contribute towards formation of a cognitive reserve, thereby preventing the effects of aging on cognition.^36^ The findings from this study are in agreement with results relating to the effects of age, in a study on performance in the subtests of oral narrative, repetition, verbal fluency, auditory comprehension (sentences), written narrative, reading, written text and sentence comprehension, in which older individuals had lower scores than younger subjects did.^37^ Like in other studies with similar tasks and batteries,^1^^,^^6^^,^^10^ age had little effect on task performance. Despite the decline in task performance found among older age groups, it has been shown that in narrative tasks the elderly have better scores and higher production of words and sentences to express pictorial information,^38^^,^^39^ thus corroborating the findings in our study. This is because the elderly tend to have more repetitions, although these are not necessarily off-topic.^40^ In speech, it is possible that repetitions are intentional, produced in order to emphasize certain information,^41^ or even to make time for organization of thought.^42^ Moreover, they may be due to the changes in communication style that occur naturally as a result of aging.

During the guided interview, age was the only predictor, contributing 3% to performance. In conversations with autobiographical topics, elderly people may have more personal observations, with frequent remembrance of the past. Moreover, with the aging process, narratives tend to have a complex plot, with more episodes and better management of resources, so as to maintain the interest of communicative interaction by making linguistic and extra-linguistic adjustments.^43^ It is important to pay special attention to the heterogeneity of communicative performance, which is greater in the elderly population.^44^^,^^45^

Some limitations of this study should be taken into consideration in order to make better use of its findings. The fact that illiterate or functionally illiterate patients were not included may have contributed towards the predictive outcome of models, with a maximum of 28% for the education level variable. Since the effect of education is not linear in cognitive tests (mainly because of lower education levels), a difference of one or two years of schooling interferes with performance.^6^ In addition, it should be pointed out that both education level and FRWH were used as quantitative measurements, thus not covering specific measurements of education quality or the quality of the material read and/or written.^18^ Thus, additional studies are needed in order to analyze these factors, so as to better understand the influence of these variables on language performance.

These data are important with regard to making correct diagnoses among patients suffering from neurological injuries, because of the potential for avoiding false positives. In addition, the variables studied are important because they establish normative data for clinical populations, especially aphasic individuals.

## CONCLUSION

These results show the influence of sociodemographic variables, especially education and its association with FRWH, among the MTL-BR tasks. Examiners should take these variables into consideration when evaluating language performance, especially in clinical populations.
